# Transferable learning from pandemic experiences: impacts, adaptations and capacity for resilience within households, communities and organisations

**DOI:** 10.1186/s12889-026-27348-7

**Published:** 2026-04-27

**Authors:** Kerry Hanna, Adele Ring, Katharine Abba, James Coleman Watson, Neil Joseph, Peter Lloyd, Mark Gabbay

**Affiliations:** 1 School of Health Sciences, University of Liverpool, Liverpool, UK; 2 Department of Primary Care & Mental Health, The University of Liverpool, Liverpool, UK; 3 NIHR Applied Research Collaboration North West Coast, Liverpool, UK

**Keywords:** COVID-19, Cost-of-living crisis, Resilience, Inequalities, Community, Health, Socio-economic impact

## Abstract

**Introduction:**

The COVID-19 pandemic continues to impact the daily lives of people and communities. The physical and mental health, financial and social effects of the pandemic, and the subsequent cost-of-living crisis has furthered existing inequalities. The pre-pandemic precarity of finances and social capital among the most vulnerable communities, has led to an exacerbation of poorer health and greater anxiety. The aim of this study was to explore how individuals, voluntary organisations and employers experienced the pandemic, and how they adapted to the changing situation and their potential relevance to challenges that have persisted since.

**Methods:**

This study is part of the COVID-LIV research programme. Semi-structured interviews were conducted with 49 participants from three societal strands: individuals/households (*n* = 27), community and voluntary organisations (*n* = 19), and employers (*n* = 6) within the Liverpool City Region. Data were analysed using thematic analysis underpinned by symbolic interactionism.

**Results:**

Three overarching themes emerged from the analysis: (1) Impacts of the COVID-19 pandemic; (2) Adapting to challenges: socio-economic and personal impact; and (3) Capacity for resilience: the pandemic and its legacy. COVID-19 and the period since have presented challenges. Closure of social spaces, changes to routine and restricted movement impacted mental and physical health. However, it was not felt equally by communities from different demographic and socio-economic backgrounds. While remote working and volunteering offered benefits, digital exclusion and burnout were challenges. Community organisations were more dynamic in meeting emerging needs, but system-wide limitations and funding constraints impacted the support available.

**Discussion:**

Findings highlight the enduring nature of pandemic-related inequalities and the compounded effects of socio-economic shocks. Structural and individual-level interventions are essential to strengthen resilience and reduce disparities. Collaborative, community-driven solutions and equitable policy measures can mitigate future crises. Lessons from adaptive responses during COVID-19 provide valuable insights for enhancing preparedness and resilience in public health emergencies.

## Introduction

During a global pandemic, decisions at individual, community, regional, national and international levels can affect the speed and spread of infection [64, 69]. Being able to limit the impact of a pandemic depends on the public health measures delivered and the existing health inequalities [10], the strength of individual and system-wide support systems in place, and the measures put in place by local and central government to address the needs of a population during times of such crises [39]. During the COVID-19 pandemic the capacity to protect one’s own health varied, depending on ability or desire to adhere to social distancing, use effective protective equipment and adequately maintain hygiene to reduce the risk of COVID-19 infection and spread [17, 28, 68]. Some groups have heightened vulnerability to infection and illness, and greater risk ill-health and mortality [18]. These include older people, some global majority ethic groups, people with pre-existing health conditions, and people who cannot shield or reduce social contact due to their job role or living in shared or crowded households [45, 63].

The COVID-19 pandemic placed greater focus on the individual actions of members of society, as well as the decisions, interventions and measures put in place by local, national and international governments [42]. Individual and collective risk impacts how, when and where decisions are made [30], and personal circumstance can influence likelihood of adhering to social restrictions and public health measures [33, 76]. Personal or familial vulnerability to illness, risk of morbidity and mortality, understanding the ramifications of the situation, and perceptions of an illness and the measures put in place, can all play a role in whether and to what degree people listen to and comply with social restrictions [3, 79].

The COVID-19 pandemic has had legacy impacts [62]. Not only is the effect of long-COVID felt by a substantial proportion of the population, but there are long-lasting mental health impacts resulting from social isolation [70, 81]. There have also been persistent effects on children and young people’s social and educational development due to a lack of interaction with others [32, 50, 56]. The financial impact following the pandemic has been far-reaching, and with the subsequent cost-of-living crisis, many families are still struggling to make ends meet [55]. The financial and social effects of the pandemic and preceding period have not been equally experienced [9]. Those underserved and underrepresented groups – including people living in more deprived areas and people from global majority ethnic backgrounds [55, 72]—have fared worse. Unemployment and debt have been evidenced thoroughly, these factors are interrelated and can cyclically impact people’s mental health [25], something also not experienced evenly across geographical, socio-economic or demographic groups [2, 4]. The unemployment, employment and job vacancies rates have only in 2025 returned to the levels seen pre-pandemic [65] However, the challenges described have not been effectively addressed by national or local government since the pandemic [22, 58]. Resultantly, it is important we understand how we can support positive, individual decision-making in relation to health and finances, and influence policy for a system that better supports people and communities facing poverty.

The COVID-LIV B study is part of a broader programme of social science research (COVID-LIV), which has explored the perceptions and risk management in relation to their impacts on individuals, households, communities, public services and employers [1, 30]. This study programme aims to describe the mechanisms and processes that influence capacity to protect population health and adherence to social distancing, hygiene and self-isolation measure for people living in disadvantaged areas within Liverpool City Region (LCR) [1, 30]. Two specific areas of this larger programme interact with this study, study areas A and D.

The aim of this sub-study is to explore the experiences and impacts of the COVID-19 pandemic and subsequent lockdowns on individuals, and how individuals adapted to the challenges and opportunities presented the pandemic. This will help to identify how adaptations and changes in circumstances impacted and potentially exacerbated individuals’ experiences, with a lens on the elements still relevant to the post-pandemic reality of ongoing adversity for many in society linked in particularly in the ‘cost-of-living crisis’ that followed the COVID-19 pandemic [24]. This study includes data collected from three separate strands within society: (I) individual/household, (II) community/voluntary organisations and (III) formal employers. Together, findings will collectively demonstrate the impacts and adaptations made in response to the pandemic, demonstrate these factors within the context of the ongoing cost-of-living crisis, and contribute to wider research as a whole.

## Methods

### Study design

This was a qualitative research study employing semi-structured interviews and photo-elicitation across three work strands and is a sub-study from a larger research project (COVID-LIV). This sub-study collates findings on the individual/household, community and organisational level, following on from recently published research in the topic area exploring how community groups and organisations responded to the pandemic [1].

### Public involvement and ethical approval

Public advisors (including PL and NJ) were involved in research team meetings, contributing to the design of the study, development of interview topic guides, data analysis, and the generation of the outputs disseminated to stakeholders and interested parties.

### Ethical approval and informed consent to participate

The study was approved by the University of Liverpool Health and Life Sciences Research Ethics Committee (Psychology, Health and Society) (Reference: 7805, 15 June 2020). All participants were fully informed about what the study involved and gave written or verbal consent for their participation and use of their data. All participants were fully informed about what the study involved and gave written or verbal consent for their participation and use of their data. This research adhered to the Declaration of Helsinki, receiving peer-reviewed ethical approval to take place, being aware of and respecting participants and their welfare and wellbeing, with all studies within the COVID-LIV study maintaining rigour and transparency and being submitted to peer reviewed academic journals.

### Consent for publication

Consent for publication was not necessary beyond the initial consent forms completed by all participants, as no identifiable images or data were presented which could compromise the anonymity of participants or participant groups.

### Reflexivity

The authors came from a range of professional backgrounds, including academic researcher, medical, healthcare, social care, applied sciences). Several of the authors are experienced in qualitative research methods, with KH, KA, MG, PL and AR conducting interviews. All authors apart from JCW were involved in coding and analysing interview transcripts. Several members of the research team have lived experience as leaders in their community and of organisations and/or were actively engaged in research as public advisors. The mixed demographic and professional backgrounds of the research team helped provide varied perspectives on interview transcripts, analysis and conclusions drawn, strengthening the findings.

### Participants and recruitment

Participants were recruited through three means. Firstly, the CLAHRC NWC – the previous incarnation of the ARC NWC—conducted the Household Health Survey (HHS). This survey asked a series of questions of people aged 18 years and over living in homes across the North West coast. Within the HHS dataset several useful demographic variables were collected, including living situation (living alone, living with children), those with caring responsibilities, people who identify as vulnerable due to long term health conditions, and those aged 70 + etc. Among a larger geographic population, the survey encompassed eleven neighbourhoods within the Liverpool City region (LCR). Survey respondents from LCR neighbourhoods who stated they were interested in taking part in future research, were contacted for involvement in this study. Secondly, snowball sampling, with ARC NWC public advisors asked to contact community members and groups that were responding to or adapting to the pandemic. Thirdly, professional networks via the CLAHRC and ARC NWC were utilised, working alongside Liverpool Health Partners, Local Employer Partnerships and other ARC NWC member organisations, including health, social care, third sector and private organisations. Refusal or lack of time/capacity to participate in the research had no negative consequences for services access or offers for involvement in future research projects. To be included in the study, participants needed to be aged 18 years or over and living/based within the Liverpool City Region.

### Data collection

This study presents findings from each of the three strands (as identified above), based on individual/household, community and organisational experiences of the COVID-19 pandemic and their responses to it, on the individual level, rather than the organisational or community level. Semi-structured interviews were conducted with 19 participants from community or voluntary organisations, 6 employer participants (a mix of both private and public sector) and 27 household participants (one of whom was also interviewed as an employer). Interviews were conducted using the topic guide relevant to the population group the participant was a member of. Topic guides were developed as part of previous research within this COVID-LIV B study [1, 30]. Interviews were conducted until the research team deemed data saturation (for interviews with individuals) or data sufficiency (for interviews with voluntary sector and other employer participants) had been met. Data saturation was considered for each strand of the data analysis framework [30] in relation to (a) the study aims, (b) the sample specificity, (c) the theoretical approach taken, (d) the quality of the discussions during interview and (e) the strategy for analysis (iterative thematic analysis).

### Data analysis

Interviews were transcribed, verified and pseudo-anonymised to facilitate contacting participants for follow-up and for linkage of different data points for individuals. Inductive coding, development and refinement of themes occurred through data immersion. Participants were interviewed twice, initially in the Summer/Autumn of 2020, with follow-up conducted in the Autumn of 2020. All transcripts were coded by a member of the research team, with a subset of 10% of those transcripts then coded by a different member of the team for validation. This helps to reduce potential researcher bias and improve the identification of pertinent themes from the data. Following review and reconciliation by the study team, a revised coding frame was developed and ordered into themes. Regular meetings informed framework and topic guide development for all three strands, ensuring consistency in interview conduct. Each participant and their interview transcript were assigned a unique, anonymised identifier indicating they were a participant representing the household, community or employer participant group, followed by the unique code number.

Thematic analysis of anonymised transcripts was undertaken using NVivo software and researchers followed an interpretive approach to analysis. The coding of transcripts and reporting of findings reflects the relative imbalances in participant numbers between groups (individuals, employers and community organisations). Consequently, with fewer participants from the commercial and employer groups, findings may more greatly identify the experiences of households and community organisations. There were frequent coding, analysis and interpretation meetings of the multidisciplinary research team, including clinicians, sociologists, psychologists alongside public advisor researchers from a range of ethnicities, social and work backgrounds. Prior to the coding of interviews, the public advisor members of the research team attended a training program on qualitative research design and theory, data coding, and methods of analysis. Thematic analysis allows the iterative generation of substantive themes and findings from qualitative data, with researchers able to identify potential biases in their interpretation and understanding of the data [11]. Our analysis was underpinned with symbolic interactionism as a theoretical framework [16], a theory that focuses on patterns of interactions between individuals within social structures and the impact of social interactions on the society, as felt by those living within it. This is therefore an appropriate lens from which to consider:

1. Participants’ perceptions of risk, adherence to risk-management directives, and the impact of directives at individual, community and organisational levels during the COVID-19 pandemic.
2. Legacy impacts on capacity for resilience in relation to socio-economic and mental health challenges post-pandemic.

## Results

### Participant characteristics

Table 1 shows the demographics of all recruited participants, further demographic information for the individual/household participants is available via the previous research published as part of this arm of the COVID-LIV study [1, 30].

**Table 1:** Participant characteristics across all three strands of research

**Demographics**	Participants from households (*n* = 27)	Participants from community/voluntary organisations (*n* = 19)	Participants representing employers (*n* = 6)
Gender	13 (48.15) female 14 (51.9%) males	11 (57.9%) female 8 (42.1%) male	3 female (50%): 3 (50%) male
Ethnicity	25 (92.6%) White British 2 (7.4%) White other)	N/A	N/A
Age (mean ± SD)	59.0 (± 13.1) [27–83] years	N/A	N/A

Research interview participants’ demographic characteristics. Gender was collected for all participants in the research study regardless of the strand group they belonged to. Ethnicity and age were collected for participants in the individual/household group. Employer and community/voluntary participants were responding as representatives of their organisations, not as individuals, as such we have not reported gender or ethnicity. Furthermore, with small numbers of participants from these sub-groups, this data was not collected or reported to ensure anonymity

### Themes and sub-themes

**Fig. 1:**
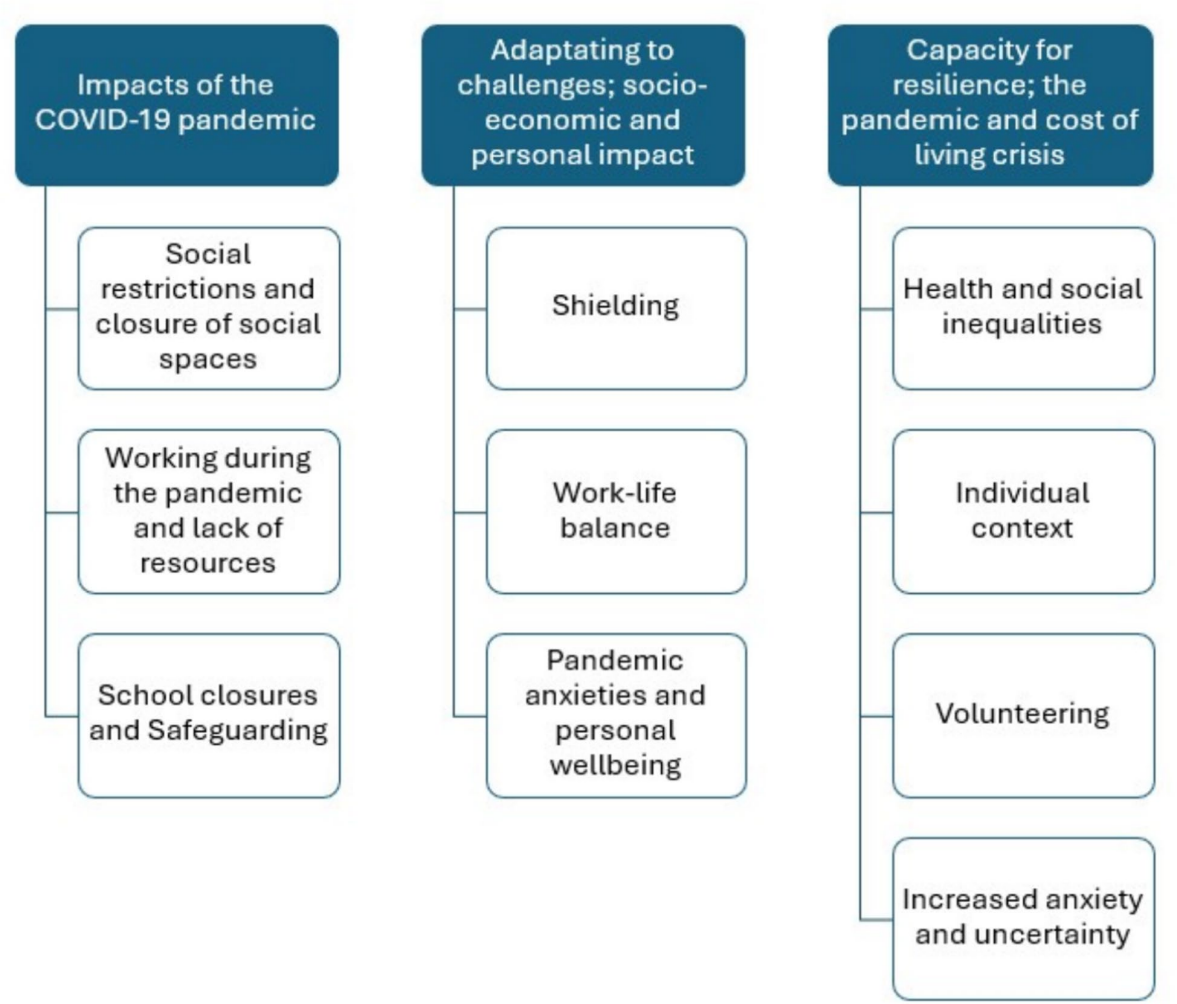
Themes and sub-themes generated from participant interviews

The findings presented here illustrate responses to the pandemic and the factors that presented opportunities, barriers and challenges to coping and adjusting, from the perspectives of individuals, and people representing their views of their organisation or community. The findings generated one overarching theme ‘*factors impacting mental health and wellbeing in the pandemic*’. Three sub-themes were identified in relation to the aims of this study specifically: (1) *‘Impacts of the COVID-19 pandemic’*; (2) *‘Adapting to challenges; socio-economic and personal impact’*; and (3) *‘Capacity for resilience: the pandemic and its legacy’* (Fig. 1). When presenting themes, pseudonyms were created to identify which strand of research the participant was recruited to: households (S1; strand 1), community and voluntary organisations (S2; strand 2) or employers (S3; strand 3), followed by their recruitment number.

#### Impacts of the COVID-19 pandemic

##### Social restrictions and closure of social spaces

Social restrictions across the UK involved sustained periods of mandatory isolation from people outside of your household. Participants identified psychological impacts from these social restrictions and limitations on social contact with friends and family, many also delved into the ongoing mental health impacts of these restrictions and social isolation generally. Although some participants identified that some people felt benefits or no change resulting from the pandemic lockdowns, they identified that the negative impacts were great and persistent.

*“...additional risks [from pandemic lockdowns] are the mental health risks to people by virtue of the isolation…I’ve got a friend who’s really antisocial who actually enjoyed it, but the point I make is it’s not necessarily all down but for some people it’s hugely negative.* [S2042]

Community and social engagement, sometimes in day-to-day activities, were lost for periods during the pandemic. The closure of community venues was referenced repeatedly by participants who identified this as a key driver for isolation for some people. This isolation was described as a catalyst for poorer mental health and wellbeing, and also impacted families lives of some people in society.

*“...all the facilities in the community...had to be stopped didn’t they because they’re the worst places...for spreading the virus. So, you know people are getting so isolated...because there’s nothing for them to do…people were feeling isolated...lonely...getting depressed.”* [S2005]

*“I think the closing of community centres meant that people...ended up being more socially isolated and I know...single parent families who...basically spent the entirety of COVID locked in a room with a small child...not able to get out...not able to see other people.”* [S2012]

### Working during the pandemic and lack of resources

There were also disruptions caused to people and businesses, personally and financially. Participants discussed the furlough system, unemployment during the pandemic, and the fallout from the furlough scheme ending and losing employment due to the impacts of the pandemic. Some even noting their reduced income impacted their ability to buy the basics, including food for their family.

*“At the start...the main needs were people who needed food basically, a lot of it was food and lack of food, a lack of funds for food…we had a lot of people who had been furloughed from work and lost 20% [of their income]...that 20% was their food money to buy food and drinks for their family so they’re now paying their bills and left with nothing at the end.”* [S2025]

During interview, participants discussed the lack of appropriate systems in place to support families whose life had been changed due to the COVID, lockdowns and financial strain.

*“I did ask last time on one of our video calls like if we [as an organisation] could try and reach more families in need] with the food because 60 [families] is nothing...there’s so many families on this estate [locally to them] struggling...and especially now with the COVID thing, people have lost their jobs and everything.”* [S2005]

People representing social support organisations discussed their desire to support more people and families who were struggling to get by, but that the demand for food and other types of support was beyond what was available. Even when most people felt the pandemic was starting to subside, some participants mentioned that financial and food poverty were still evident and a real issue that many were still facing daily and having to make choices over what bills to pay and who was eating.

*“...although people will say ‘well we’re moving to recovery phase’ [post-pandemic], [it was] not [the case] for a lot of people, [they] were still very much still in response, you know people are still looking for food parcels, people are...being made redundant now that the furlough schemes ended.”* [S2001]

*“We had people who, because the children were home, couldn’t feed the children because normally they’d be on free school meals...So, we were giving those families...the minimum of 3.5 meals a week so it meant that half of their meals were seen to, their main meals… anxiety was very high, parents were so worried about their children...the parents weren’t eating...they were looking to the children’s needs first.”* [S2040]

### School closures and safeguarding

To reduce the risk of COVID-19 spread schools were closed for substantial amounts of time. Cross-generational exposure was identified as an important factor among participants, with some discussing interactions with their grandchildren leading to illness and children contracting COVID-19 when they were in school. Limiting the risk of initial infection and the spread of infection as important, particularly for people who lived or had contact or lived in households with older people, or people who were more vulnerable to negative health outcomes due to pre-existing health conditions.

*“But my own experience was my family and particularly my young grandson and the whole of his school had a cough for quite some time, and I can distinctly remember him coughing right in my mouth…and I got ill after that”* [S1006]

*“I’ve been going to work every day throughout, I use public transport, I have young children in school, so yes I think there’s a reasonable risk that I could contract it [COVID-19], or I may have already contracted it.”* [S1015]

Although efforts were made to implement remote measures for teaching and supporting children to learn, this was all done quickly with limited time to plan, and so many children were faced with restrictions to their education and development. Existing inequalities were also exacerbated. Some households did not have access to technology to facilitate remote learning, and some lived in homes with multiple children, restricting their access to the technology and environment to learn.

*“My main observation has been that this has digitally excluded groups who can’t get online so yeah, I don’t know who those groups are, but they’re not groups I work with…well if you can’t afford food, you can’t afford WIFI either.”* [S2012]

*“It [home schooling] was it was quite challenging because as I say the mix of the children who could access online learning and the mix of the children who couldn’t was roughly about maybe 60% that could get it online, so it was quite tricky for staff.”* [S32003]

The terms of school closures differed across the country and the ability of schools to move online and provide teaching and learning for pupils also varied. School closures impacted children and teachers, as well as parents and guardians, with the frequent closing and re-opening of schools affecting parents’ and guardians’ ability to effectively organise childcare.

*“I know there’s been challenges definitely in [Region 1] about school years going home for 2 or 3 weeks at a time...it’s been really challenging for the kids and we’re seeing it when we come back. Not only from a mental health point of view but, you know, kids of that age...exercise almost needs to be part of their daily routine”* [S2004]

Closure of spaces, including schools had an impact on the potential for identifying safeguarding issues and supporting people through emerging and changing needs. The lack of face-to-face contact, and limitation of opportunity to talk to people who may be vulnerable or have acute support needs or observe their home-life for instance was identified as an ongoing pandemic challenge. One participant in particular noted that women in abusive relationships or at-risk of domestic violence at home may have lacked any support during this period due to the social restrictions. Another participant highlighted the lack of social spaces frequented by older people which could result in a like of safeguards being put in-place, or support being offered to them.

*“we [community support organisation] had...women who were in domestic abuse relationships or who were at risk who did not feel they could leave [their homes] because of the [social] restrictions or did not feel that services would be able to support them because of the [social] restrictions.”* [S2034]

*“...it’s removed all of the safety nets, so the spaces where parents could go, the spaces where older people could go, the libraries all of these things have all disappeared so there is no safety net now in there for people to get any support.”* [S2011]

School closures and intermittent face-to-face teaching also presented issues with safeguarding of pupils. Teachers had less time to talk to children about potential emotional or other issues. Some participants discussed the challenges and ramifications of this, as well as the fact there were limited public spaces for families to go to in order to discuss these challenges and engage with services.

*“There’re kids like that obviously [live] in abusive families...when they were off school as well… they weren’t getting identified in school. Whereas when they’re going to school they’re getting looked at...checked on and looked after and monitored type of thing.”* [S2005]

*“...it’s removed all of the safety nets, so the spaces where parents could go, the spaces where older people could go, the libraries all of these things have all disappeared so there is no safety net now in there for people to get any support from.”* [S2011]

The lack of safeguarding in-place and capacity and availability of providing safeguards was also discussed when thinking about vulnerable and older people. The lack of face-to-face contact with people who may require additional support, some felt could lead to struggles in identifying concerns any additional or changing support needs.

#### Adapting to challenges; socio-economic and personal impact

##### Shielding

There was a myriad of challenges presented to all age groups, but more acutely felt among some individuals and communities. Those who were vulnerable to infection and ill-health from COVID-19 were advised to shield, remaining at home and limiting social contact to reduce their risk of infection. Many participants discussed not only the practicalities of shielding when in a multi-generational household, or a household reliant on some family members’ income, but also the reduced social connection and poorer mental health that people experienced when shielding.

*“...when I was shielding I was proper terrified in the house because I wasn’t on the outside and I didn’t know what was going on and my son had to go out to work because his job was one of them jobs where you had to…every time he come back in it was like ‘wash your hands...where have you been?’...And I had to say in the end ‘you don’t realise what it’s like for me in here, because you haven’t got a clue what’s going on outside’* [S2005]

*“Predominantly it was about wanting to be connected to people...exacerbated by the fact that they [people shielding] couldn’t leave the house...as an example, you’ve got over 70 s [years of age], you had people with health issues, so we have people [in their community] that...were not supposed to be leaving the house...”* [S2028]

##### Work-life balance

Some organisations and professions were able to be dynamic and provide their service online. Working remotely was seen as a positive for many people, reducing time travelling and money spent on commuting, and removing the stress involved in travelling for work. The move online helped to provide an easier day-to-day and establish an improved routine, allowing people to spend more time with their family, as well as providing secondary financial and climate-related benefits.

*“I see real possibility for reorganising the way people do work...I’ve just signed up for a job in Cardiff, but you know I can do it perfectly well online and that saves the environmental damage of me travelling, not to mention those who do long commutes every day at huge costs to themselves as individuals*.” [S2042]

*“I had a two-hour commute into the office and a two-hour commute back. So for me, it's been great because even though I've done more [meetings] on Zoom and Teams I’ve cut out all that traveling time, and most of the teams have said the same...as well as the commute, they’re cutting out driving around the patch [local area], which is stressful and busy...So even though we're doing more [work]...getting more done in the day...we've got a bit more balance, because we're not doing the traveling.”* [S3002]

However, in moving their job online in order to reduce the risk of COVID-19, some felt social isolation was impacting wellbeing due to reduced social interaction. Some organisations and businesses also discussed the impact of social isolation on employees’ mental and physical wellbeing.

*“The adaptations and changes we’ve had to make to our working patterns [by working remotely] and also our home life...is a direct consequence of isolating ourselves from the virus...It’s [changes to working practice] also about the wellbeing element of it...people are not socialising at the moment...We sent a lot of people home...certainly those who were furloughed who lived by themselves...we knew that they were going home and we knew that a lot of these people where you know lived by themselves.”* [S31005]

##### Pandemic anxieties and personal wellbeing

Finding ways to maintain positive mental health and wellbeing for people during the COVID-19 pandemic presented a challenge. Many discussed issues with their own and other people’s mental health, due to the anxieties surrounding the risk of COVID-19, not being able to visit family and friends, having to maintain social distancing, and not being able to live their ‘normal lives’.

*“I can’t help but feel that we have a bit of a mental health storm coming as well because I think people have become insular, very anxious as I say some groups of people have become even more isolated than they previously were. Because [previously] where they had a space that they could go...find companionship and cohesion [that] has disappeared…”* [S2012]

Some people felt that their, knowledge and expertise around health and research, and their existing health literacy supported them in understanding how to navigate public health restrictions, and the additional measures needed to stay safe, for example one participant mentioned *“when I visit my friends they’re all sort of careful you know, put the hand gel on *etc.*”* [S1005]. Social restrictions caused a great deal of anxiety and stress amongst the population and was heightened as the pandemic and limits on social movements lasted longer than many imagined it would. One participant identified the stress that came from trying to maintain social distancing, but understanding the wellbeing benefits of personal, social interactions.

*“sometimes say like a neighbour’s kids will run up to me and just come for a hug...which I know is not within the letter of the guidelines but I’ll just kind of do that because its humane… you can kind of do the mental maths…[but] the elderly, it’s just not fair to them to put them in any risk”* [S1026]

#### Capacity for resilience; the pandemic and cost of living crisis

##### Health and social inequalities

Participants identified that there was a lot of individuality in how people could and did respond to the pandemic and subsequent lockdowns. Informal, emotional support, through family and friends was essential to many, but there were situations in which this support was not available. This resulted in distress for both those in need, and those who would normally provide support.

*“we’ve got 2 friends who’ve lost partners in this period [COVID pandemic], and they’re absolutely devastated that...none of us can go round and see them...your automatic response when a partner dies you...support them...But both of those elderly people are desperately lonely...ringing up for an hour at a time... But for them their mental health is so damaged because...there’s not that...support network for when they’ve had somebody die.”* [S2040]

Participants also discussed the greater financial and social burden experienced by some members of society. People were not always able to work remotely, with some people having to go into work every day, often using public transport and coming into contact with colleagues and members of the public. Some professions – such as health and social care workers, or supermarket staff—were confronted with situations which impacted their ability to avoid risk of contracting COVID-19, as a result of the decisions made by management and employers regarding PPE.

*“For months we didn’t have face coverings...my boss was aware that the Government advice was that they weren’t compulsory, so he didn’t get us them, then eventually that seemed to change, and he did get them. But then never gave us all direct guidelines on when we’re meant to wear them...even though we’ve got them most of us aren’t wearing them”* [S1003]

Some mentioned that inequalities existed previously, with areas that were historically underfunded facing heightened and more complex social, logistic and financial issues during the pandemic. However, these inequalities were brought more into focus during the pandemic.

*“...I think a lot of the working policies and everything else is very London-centric and it doesn’t take into account the difficulties that we have particularly in the North and North East due to years...of un-investment in our services and the fact that we are starting from a lower point in terms of our health and inequalities in the first place. Which means something like this hits us harder which is why we’ve seen higher figures for deaths etc”* [S2012]

Financial issues were presented as a result of social lockdowns and the closure of public services. Participants working to support people living in their communities identified that maintaining employment and or, receiving welfare benefits became a constant challenge. For families whose children had received free school meals, school closures meant these meals were not available, so finances shifted in order to provide meals for their children, leaving less money to pay bills and for parents or guardians to feed themselves.

*“We had redundancies and those families. We had people who because the children were home [the parent(s)/guardian(s)] couldn’t feed the children because normally, they’d be on free school meals, and they were finding it really hard.”* [S2040]

*“... at least then [IN SCHOOL] when they were going the breakfast club they were getting fed and, you know, had a little full stomach for the day…and the [food] vouchers, people weren’t getting them for 2 to 3 weeks...they needed them then immediately…by the time they’d got out [received the food vouchers]...kids probably would have starved to death.”* [S2005]

Participants discussed the fact that people who had not previously been in as much need, were suddenly requiring support, with systems failing to account for these families, it resulted in delays.

*“We normally do a summer hunger programme anyway to support families who essentially would normally be getting free school meals...because of COVID a lot of families suddenly found themselves out of work or not registered for free school meals. So, they just fall through the gap...they weren’t given free school meal vouchers or anything...So that's when we stepped in to fill that gap...we do three days a week, we provide a packed lunch and activity packs to families who are registered with us.”* [S2027]

As the UK moved into a ‘recovery’ phase post-pandemic, with the opening-up of services, participants still felt that many were enduring the impacts of the socio-economic issues that COVID-19 and the national response to COVID-19, had instigated.

*“You know although people will say ‘well we’re moving to recovery phase’, not for a lot of people, we’re still very much still in response...people are still looking for food parcels, people are being made redundant now that the furlough schemes ended.”* [S2011]

##### Volunteering

Although some participants volunteered at local social organisations or charities prior to COVID-19, adapting to the pandemic for some meant taking up volunteer roles. This often came about through a desire to help others, and to remain active due to a termination of existing employment during the pandemic. However, the stress and anxiety felt in community-facing roles was evident among volunteers. Often coming into contact with members of the public, not only were they witnessing enduring hardship in their community, but they were also at greater risk of contracting COVID-19, and anxiety associated with a lack of social distancing and contact with others.

*“...there was a lot of people who…because the pressure that was placed on them [in their volunteer role] there was big concerns about fatigue and people burning out. A lot of people worked incredibly hard at the [Organisation 11]...doing 12-hour days regularly.”* [S3005]

People who volunteered during the pandemic felt a sense of pride in their work. Participants discussed the benefits they felt from volunteering, both during and after the pandemic, emphasising its importance in helping people in need.

*“I keep in contact with all that group of people [volunteers]...I ask them ‘how things are going?’ They’re saying that they’ve got as much out of it as the people who they’re supposed to be supporting, so it’s been incredibly successful.”* [S2026]

##### Increased anxiety and uncertainty

The changing evidence, physical and health anxieties and structural inequalities presented challenges and uncertainties for many. Participants discussed their worries around the ‘opening up’ of society following periods of lockdown and the lack of thought going into these decisions.

*“I think the Government is far too keen to get people out and spending again for political reasons and not keen enough to encourage people to be cautious”* [S1022]

Other participants noted that the uncertainty around COVID-19 and social isolation has persisted throughout. The continued impact of COVID and the associated stress on people and communities was discussed by a participant who took part in a second interview.

*“I feel there’s a lot of anxiety amongst community groups...as to how they’re going to manage this [impact of COVID-19] …I don’t think it’s a health risk, it’s more about the social impact”* [S2012]

The continuing confusion and worry about whether some people would be able to see family and friends, particularly older relatives living in care homes, and the extent to which this was impacting their mental health was noted amongst several interviews. The constant worry about employment, work, schools and other factors were also affecting wellbeing and presented coping challenges. This was particularly the case for women, as noted by a participant from a community organisation.

*“They don’t know one day from the next whether they’re going to be able to see their family...to visit care homes, if their children can go to school, if they can go to work. You know as much as the rules are clear, it doesn’t make it any more certain in respect of people’s individual realities...”* [S2034]

For some participants, the present anxiety issues were discussed, but some also mentioned worries about the future, particularly the social restrictions and potential financial and social changes that could come with further upheaval. When asked of the challenges, one participant stated.

*“Simply the uncertainty...what is it going to be like. What will I be able to do in a week’s time, 6 weeks’ time, a year’s time? [This] is of itself hugely stressful because it restricts people’s opportunities, their ability to maintain social structures is compromised.”* [S2042]

The uncertainty around COVID-19 and how long it would last, impacted on people’s mental health. Being able to see the end of the period was a focus for some, and with the pandemic and societal changes continuing, many started to feel a greater burden and struggled to cope with the mental health challenges this presented.

*“...we are going to be mask-wearing for at least a year if not two, if not, you know, we don’t know…I started this thinking ‘oh it will be a month’ and it’s like it’s September. I think that’s why I’m having a bit of a wobble because it’s like ‘how did we get to September?”* [S2028]

## Discussion

This study brings together the experiences, and both personal and societal impacts felt by people living and working in Liverpool during the COVID-19 pandemic. Interviews with participants have highlighted the physical and mental health effects of both COVID-19 and accompanied social restrictions, as well as the wider effect of these factors on social cohesion, the exacerbation of the wider social and systemic determinants of health, social capital and societal resilience. The findings in this study provide points that remain of significance given the persisting financial, social and health-related impacts that have stemmed from the cost-of-living crisis which followed the pandemic. Interviews highlighted the variation in capacity and desire – both individually and societally – to adhere to social restrictions, whether due to the nature of employment or pre-existing health conditions which put them at risk of COVID infection and the impacts of the virus, or personal choice [1, 30]. Participants demonstrated the negative impacts of COVID-19 and the pandemic, especially the impact on employment, finances and the mental health and wellbeing due to ongoing stress around COVID infection and impacts on social interaction. The financial and social impacts were not felt equally, with some groups encountering greater challenges, whether that be in their employment, finances or health, which is also identified in existing research [9]. These are impacts which have persisted in the years following the pandemic, with those already disadvantaged groups enduring greater financial uncertainty and a lack of support [38, 55]. Although the findings highlights that social changes and restrictions gave some the opportunity to support others and gave them a sense of purpose during a difficult time, many felt that this came about due to a lack of jobs or appropriate financial support for people out of work. Additionally, some felt the system was overwhelmed, and local and national governments were not capable of looking after the most vulnerable in society. The systemic issues in community support have persisted post-pandemic, with many experiencing issues with physical and mental health due to the now day-to-day nature of anxiety which has been exacerbated by challenges in accessing employment and finances [6, 53].

### Inequalities, resilience and supporting change in work and life patterns

The pandemic necessitated changes in behaviour patterns and expedited the ways in which individuals and organisations run and function [27]. Those who were able to, worked remotely for sustained periods, with the pandemic and social restrictions hastening a change in working practices [61]. Although people experienced some positive impacts of remote working, the changes to routine and a lack of work-life balance did have negative impacts on mental health and wellbeing [37]. Some professions were not able to work from home and were at greater risk of COVID-19 infection [75]. Also, ‘key workers’ often experienced acute anxiety and worry due to their increased exposure to potential infection [52]. Those working in frontline social care and healthcare roles, were more likely to be living in areas of greater deprivation or be from global majority ethnic groups [35]. Additionally, people in frontline caring roles from global majority ethnic groups were less likely to have the appropriate protective equipment necessary to reduce their risk of infection [35]. These factors further identify the variation in risk exposure and health impacts of the pandemic. Continued lack of investment in public services, furthered by the economic consequences of the pandemic have exacerbated existing inequalities which have persisted since, with underserved and underrepresented groups continuing to experience social and financial challenges [5, 12].

### Social cohesion and social inclusion

During the pandemic, there were considerable changes to the way people worked [36], and the way society interacted [47]. Being isolated and limited social contact for sustained periods has altered the willingness and desire of some people to interact with others, as well as developing greater anxiety and stress in social situations [40]. Interviews in this study have highlighted the way in which, over a relatively short time period there was a change in people’s willingness and capacity to engage in social activities, even those day-to-day activities such as going to the shops, which may have been taken for granted pre-pandemic. The isolation and associated anxiety were particularly evident among school children and older adults [48, 51]. Children were no longer in a position to meet up or talk with their peers, which existing research demonstrates has had a profound impact on their capacity in preceding years [41, 44]. People shielding due to existing health conditions, including care homes residents, went for sustained periods of time without seeing family or friends [21]. Isolation impacted mental wellbeing, with acute health impacts and exacerbation in symptoms and faster progression in those with more acute needs, such as people with dementia [26]. The restrictions on social contact led to a perceived lack of safeguards to support people and communities. This was exemplified for children who were not attending school, and for vulnerable adults and older people, particularly those in care or with additional needs. People were not seen as frequently as previously, and the lack of in-person contact made it difficult for appropriate safeguards to be put in-place, or for changing needs to be identified. This aligns with the existing evidence from the pandemic and highlights the necessity of some services to be provided face-to-face, and hybrid or remote service interaction to take place when appropriate and necessary [60].

The pandemic brought about a sense of isolation for many, there were also positives identified from the pandemic. Some reflected positively on the part they played as volunteers. Although it was felt that the provision of food parcels and other necessities should not be reliant on charitable community organisations, people felt it gave them the opportunity to ‘give back’. This example of social togetherness enabled a sense of pride in supporting others in their time of need. The existing evidence indicates that beginning and maintaining volunteering during the pandemic was more likely among some socio-demographic groups [20]. They were more likely to be female, have higher incomes and educational qualifications [49]. People living in more deprived areas, or from global majority ethnic backgrounds were more prone to food and financial insecurity during the pandemic [19], [73]. These factors presented more pressing concerns and would likely mean that people already struggling would not have the time or capacity to volunteer [49]. Greater support for disadvantaged groups, reduction of food and financial insecurity can support a more equal society, reduce strain and anxiety [8, 57]. By narrowing inequalities, addressing the systemic and financial factors associated with lower social or financial capital, we can provide more opportunity and capacity for involvement in volunteering for underrepresented communities [71].

### Employment and continued impacts of COVID

Health and social inequalities existed in the UK and globally prior to the COVID-19 pandemic [74]. However, the pandemic brought this into greater focus and furthered the inequalities between people and population groups with already varied access to services and health [23]. The impacts of COVID-19 [78] and subsequent cost-of-living crisis have resulted in sustained financial issues. This resulted in those people who were already disadvantaged needing to make difficult decisions to fend off food poverty [77, 80]. The UK Government provided financial and social support during the pandemic, and in the time since, but this has been reactionary and not addressed the factors behind the wealth inequalities and drivers of poverty [67].

The findings presented here identify that during COVID-19 and potentially since, the capacity for resilience at the population and systemic level is limited [34]. Anxieties around finances and the basics of life are now more persistent, resulting in the exacerbation of poor mental health [12, 43]. Again, this has been felt more greatly among already disadvantaged and underserved communities, particularly those already facing poverty prior to the pandemic and cost-of-living crisis [15]. It is important to address structural and individual inequalities to reduce the gap between those who have the social and financial capacity to be resilient in crises, and those who are disadvantaged by a system that is not designed to work fairly. This can be achieved through improved welfare support, greater employment opportunities, increased involvement of community support groups, better pay, and improved terms for workers [46, 54].

With a proportion of businesses and voluntary organisations failing during and since the pandemic, it is noteworthy that charities and community organisations were more able to adapt than commercial organisations. These shifts have societal consequences and costs. For example, loss of commercial rents and business rates diminishing local government income, alongside reductions in real-terms central government funding for local authorities can have impacts on finances available to provide support and community activities [7, 59]. The findings presented here suggest the voluntary sector was dynamic and adaptable, but fewer people volunteered as the pandemic ended. Communities can benefit from rediscovering communal solutions to providing support in crises and reflecting on these events to provide improved response and capacity for effective support in future crises [29, 66]. The pandemic demonstrated adaptability and motivation within households and communities, but doing so needs more effective application to enable strengths to align with household and community needs.

### Limitations

This study did not have ethical approval to do post pandemic follow-up. Although we consider the data we collected is relevant to the post pandemic adjustments, we do not have confirmative data that the changes and adaptations made by our respondents have continued, or the ways in which people, groups and organisations have evolved since. However, our data do show the ways in which adaptations were made across three societal levels—household, community organisations and formal employers—to meet the changing circumstances through the pandemic period. Many of these dynamic factors remain relevant, and in some cases, are even more acute felt in the post-pandemic environment for people and organisations with limited social capitol and assets. The emerging Covid Enquiry reports have shown how strategic decisions and judgements at the centre of government had significant impacts on morbidity, mortality and the day-to-day lives of individuals and communities such as these [13, 14].

All research participants from the participant sub-group for individuals were from White ethnicity groups. This does not fully reflect the regional demographics where the study took place. There was local anecdotal evidence that migrant populations were particularly wary of participating in research, and we recognise that as a limitation of this dataset and report. However, we did set up a separate project published elsewhere that focused specifically on the Muslim community and covered much of the same topic areas as existing evidence and research conducted [31].

## Conclusion

The findings presented demonstrate the impact the ongoing impact of the COVID-19 pandemic on individuals, employers and voluntary/community organisations. With the impact of the pandemic, and financial crises since, different groups were more vulnerable to the financial, health and societal impacts that have persisted since COVID-19. Equally, the evidence demonstrates variation in the capacity of people and groups to adapt and respond to crises, whether that be to the event, or to the financial, health-related or social impacts. Our findings and existing literature highlight that many people turned to volunteering during COVID-19 out of a sense of duty, or due to a change in employment circumstances, but the volunteer workforce was not representative of the communities served. Additionally, disadvantaged communities were restricted in their capacity to adapt and respond to the pandemic, and the social and financial challenges it brought. To address these imbalances, greater support needs to be provided for disadvantaged communities to reduce economic insecurities, anxiety, and provide avenues for a more representative volunteer workforce.

## Data Availability

The datasets generated and/or analysed during the current study are not publicly available due to ethical requirements to maintain anonymity but are available from the corresponding author on reasonable request.

## Abbreviations

ARC NWC: Applied Research Collaboration North West Coast
CLAHRC NWC: Health Research Collaboration for Leadership in Applied Health Research and Care North West Coast
COVID: Coronavirus Disease
COVID-LIV: Coronavirus Disease-Liverpool
HHS: Household Health Survey
LCR: Liverpool City Region
LSE: London School of Economics
SD: Standard Deviation
UK: United Kingdom
